# Primary hepatoid carcinoma of the ovary: A case report and review of the literature

**DOI:** 10.1097/MD.0000000000020051

**Published:** 2020-05-08

**Authors:** Won-Ku Choi, Dong-Hyu Cho, Chang-Yeol Yim, Na-Ri Lee

**Affiliations:** a Department of Obstetrics and Gynecology, Jeonbuk National University Hospital-Jeonbuk National University Medical School; b Research Institute of Clinical Medicine of Jeonbuk National University-Biomedical Research Institute of Jeonbuk National University Hospital; c Department of Internal Medicine, Jeonbuk National University Hospital-Jeonbuk National University Medical School, Jeonju, Republic of Korea.

**Keywords:** alpha-fetoprotein, hepatoid carcinoma, ovarian cancer

## Abstract

**Introduction:**

Hepatoid carcinoma of the ovary (HCO) is a rare disease that originates from the ovarian surface epithelium. It is histologically characterized as hepatocellular carcinoma (HCC) with a hepatocyte-rich granular cytoplasm.

**Patient concerns:**

A 65-year-old female patient was admitted with complaints of indigestion, abdominal bloating, and pain.

**Diagnosis:**

The patient showed an elevated level of serum alpha-fetoprotein (AFP) with abdominal bloating and pain. After surgery and histopathology analysis, she was finally diagnosed with HCO, Figo stage IC.

**Interventions:**

After cytoreductive surgery, she underwent adjuvant chemotherapy with carboplatin and paclitaxel. Although the disease was diagnosed at an early stage, it recurred 6 months after completion of adjuvant chemotherapy. Elevation of serum AFP level and removal of a mass from the lumbar vertebra confirmed the recurrence of this disease. Subsequently, the patient underwent radiation therapy and palliative chemotherapy.

**Outcomes:**

She died 31 months after the diagnosis due to disease progression.

**Conclusion:**

The aggressive nature of HCO was clearly observed in this case despite early diagnosis and treatment. Further studies are needed to understand the proper treatment and prognostic factors of HCO.

## 1 Introduction

Hepatoid adenocarcinoma is a carcinoma with hepatoid features that occurs in organs other than the liver. It has been reported most commonly in the gastrointestinal tract, especially in the stomach. Other organs include the lung, bladder, kidney, uterus, fallopian tube, peritoneal cavity, and ovary.^[1–5]^

Hepatoid carcinoma of the ovary (HCO) is an extremely rare histologic subtype of ovarian cancer. Only a few cases of HCO have been reported.

Treatment for HCO and epithelial ovarian cancer is similar and includes cytoreductive operation and chemotherapy (carboplatin and paclitaxel-based therapy).^[3]^ Nevertheless, the histogenesis of HCO has been controversial. Recurrence of HCO is common, and it often has a limited response to chemotherapy. Therefore, it is necessary to establish diagnostic and prognostic markers for HCO and an optimal treatment regimen based on histological and molecular biological studies of HCO cases.

Herein, we report a case of HCO in a 65-year-old woman. The HCO was diagnosed relatively early, and adjuvant chemotherapy was performed after tumor removal. However, recurrence in the lumbar spine resulted in the removal of the mass, postoperative radiation therapy, palliative chemotherapy, and finally, death due to intraabdominal metastasis. We describe the 31-month-long treatment of the patient.

Characteristics, treatment, and prognosis of this disease are described here along with a review of the literature.

## 2 Case report

A 65-year-old female patient was admitted to our hospital with complaints of indigestion, abdominal bloating, and pain that started a month earlier. She was diagnosed with hypertension a decade ago and was taking calcium channel blocker. She had undergone hysterectomy for a myoma 15 years ago. There was no other history (surgery history or family history of gynecological diseases). Noted signs of vitality were normal at the time of admission. On physical examination, she had abdominal distension without tenderness and rebound tenderness and no hepatosplenomegaly. Laboratory analysis showed normal liver and renal function including complete blood count. There was no abnormality in hepatitis test including hepatitis B. Serum levels of alpha-fetoprotein (AFP) were increased to 83,164.6 ng/mL (normal range, <8 ng/mL). Moreover, the serum cancer antigen (CA)-125 level was increased to 340.8 U/mL (normal range, <30.2 U/mL). However, levels of carcinoembryonic antigen, CA-19–9, and human chorionic gonadotropin were within the normal range. There were no abnormal findings on the upper gastrointestinal endoscopy and colonoscopy.

**Figure 1:**
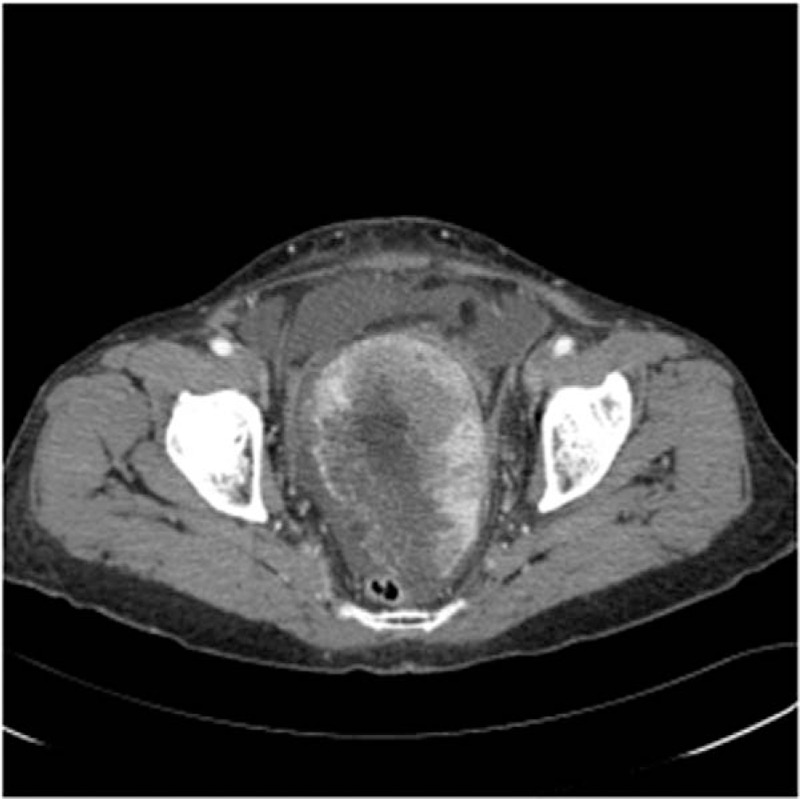
Abdominal CT scans for the patient. CT scan showed massive ascites with a pelvic mass measuring 12 × 7.8 × 8.2 cm. The pelvic mass consisted of multi-lobulated, cystic, and solid contents.

Based on pelvic and abdominal ultrasonograms, a large number of ascites in both paracolic gutters and a 13-cm mass of inhomogeneous echo were identified. Computed tomography (CT) of the abdomen showed a 12 × 8 cm sized mass composed of solid and cystic tissues (Fig. 1). There were no abnormal findings in the liver, bladder, cervical stump, or colon.

A malignant ovarian tumor was suspected, and diagnostic laparotomy was performed. A 13-cm mass was identified near the left ovary. It was encapsulated by the membrane and adhered to the sigmoid colon and cul-de-sac. On the cut surface of the mass, a mixture of yellow and white solid and cystic tissues and necrosis with hemorrhage were observed. A large number of ascites was observed. However, no nodule was found in the abdominal cavity, including the anus. Liver and diaphragm showed no abnormal findings. Frozen sections sampled during the operation showed a poorly differentiated adenocarcinoma.

**Figure 2:**
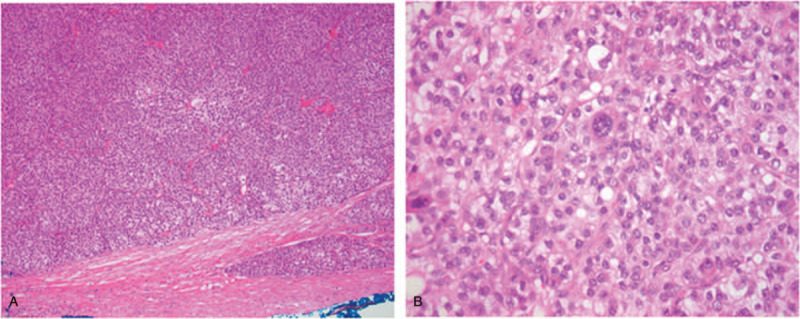
Light microscopic examination. (A) Tumor cells were polygonal and oval-shaped under light microscopy. They were separated by fibrous septa and composed of solid sheets of cells with distinct borders (HES × 100). (B) Tumor cells had moderate amounts of eosinophilic cytoplasm. Their nuclei were located in the center of cells with prominent nucleoli. Bizarre giant cells with gigantic nuclei were seen sporadically (arrow) (HES × 400).

**Figure 3:**
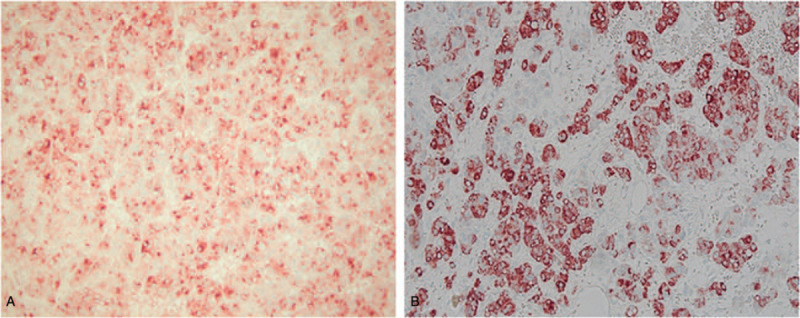
(A) and (B) Immunohistochemistry for alpha-fetoprotein (AFP) and hepatocyte-specific antigen (HSA). Tumor cells are immune-reactive to AFP and HSA (immunostaining × 200).

Histologically, the mass was ruptured and divided into fibrous septa and necrosis in the center. It was composed of polymorphic cells arranged in a solid or trabecular pattern, similar to hepatocellular carcinoma. At high magnification, abundant eosinophilic cytoplasm and nuclear pleomorphism were clearly observed (Fig. 2). Immunohistochemical staining was positive for AFP and hepatocyte-specific antigen (Fig. 3). The histological examination did not find any cancer cells in the sigmoid colon and ascites. Based on these results, the patient was diagnosed with hepatoid carcinoma, stage IC (FIGO, 2014) that originated from the ovary.

After surgery, adjuvant chemotherapy was administered with carboplatin (AUC, 5) and paclitaxel (175 mg/m^2^). After the third cycle of chemotherapy, serum levels of AFP and CA-125 normalized to 5.9 ng/mL and 7.1 U/mL, respectively. There was no recurrence or metastasis detected in imaging studies performed after the third and sixth cycle. However, 6 months after the completion of chemotherapy, the patient was suspected of having recurrence based on elevated serum AFP level of 8872.4 ng/mL. ^18^Fluoro-2-deoxy-D-glucose positron emission tomography/computed tomography (^18^F-FDG PET-CT) was performed, and FDG-uptake mass was observed in the lumbar spine (L2). Magnetic resonance imaging (MRI) of the whole spine was performed for accurate diagnosis that revealed a fracture with a mass protruding from the lumbar spine (L2) on the left psoas muscle. She underwent an operation for mass removal and posterior stabilization with Perfix system. A complication during the operation was massive bleeding from the mass. Biopsy and histological examination of the lesion site confirmed the diagnosis of metastatic hepatoid carcinoma. After surgery, AFP decreased to 2317.6 ng/mL. The patient refused further treatment due to weakness and pancytopenia. After 3 months, AFP again increased to 5526.9 ng/mL. The patient underwent external beam radiation therapy to the lumbar spine (L2) area. After radiation, AFP decreased to 1776.0 ng/mL. However, after 2 months, AFP again increased to 4726.0 ng/mL. CT of the abdomen revealed multiple enhancing nodules in the pelvic dependent portion. The patient received palliative chemotherapy with carboplatin (AUC, 4) and gemcitabine (1000 mg/m^2^). After two cycles, abdominal CT revealed multiple, newly developed, intraabdominal metastatic nodules with a serum AFP level of 12,254.9 ng/mL which was rapidly increasing. She refused further chemotherapy due to a sudden decrease in performance. The patient continued to undergo symptomatic treatment such as ascites and pain control. She died 31 months after diagnosis due to disease progression.

## 3 Discussion

Ovarian cancer is the tenth most common cancer among Korean women, accounting for 2.4% of all female cancer cases. By age, those in their 50s had the highest percentage (38.4%) of cancer incidence, followed by those in their 40s (21.7%) and 60s (17.4%).^[6]^ In 2017, 2618 new cases of ovarian cancer had occurred, and the incidence of ovarian cancer tends to increase every year. Of the different types of ovarian cancers, HCO is very rare. Only 15 cases of HCO have been reported over the 10-year period.^[3,7–19]^ Their clinical features are presented in Table 1.

**Table 1:**
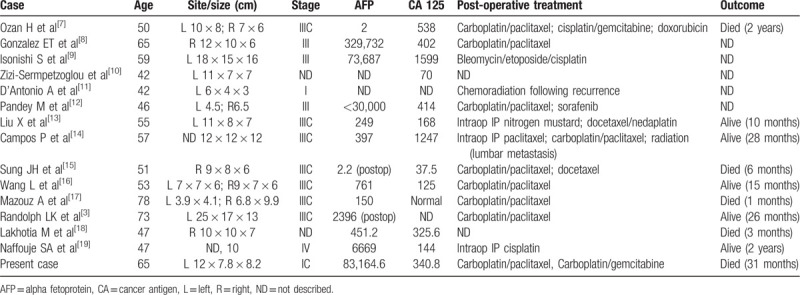
Clinicopathological features of HCO.

In all case studies including this case, the size of the ovarian mass was >10 cm in 66.6% (10) of cases. It was found to be unilateral or bilateral. Abdominal pain was the most common symptom of patients at the time of diagnosis. In addition, there are cases of nonspecific symptoms due to gastrointestinal disturbances such as abdominal distension, abdominal mass palpitations, and anorexia. Although it is known to occur in older women aged 34 to 78 years old, there are cases in which women in the reproductive age were affected. At the time of diagnosis, serum levels of AFP and CA-125 were elevated in most cases (10/13) except for three cases where AFP and CA-125 were in the normal range. The stages in which HCO was identified were stage I, 15% (2 cases); stage II, 7.6% (1 case); stage III, 69% (9 cases); and stage IV, 7.6% (1 case). The majority (76.6%) of patients were diagnosed at an advanced stage (stages III and IV).

HCO is a carcinoma that is characterized by a high serum level of AFP with hepatocellular differentiation and is positive for cytokeratin on immunochemical staining.^[20]^ It is commonly accepted that ovarian cancer has an epithelial origin due to its high incidence in postmenopausal women and a typical immunochemical staining pattern. However, an alternative theory of germ cell origin has been suggested.^[3]^ Thus, the histologic origin of ovarian cancer remains controversial. In addition, hepatoid adenocarcinoma has been reported along with carcinomas of other subtypes (serous papillary carcinoma, mucinous/serous/endometrioid adenocarcinomas, and Sertoli-type sex cord-stromal tumors), although it develops independently in most cases.^[11,13,21,22]^

Histologically, HCO is similar to HCC. It has a clear boundary with cuboidal tumor cells arranged in trabeculae or sheets. Non-metaplastic cell division is commonly observed. Furthermore, it contains a hyaline globule inside or outside the cell with an abundant eosinophil cytoplasm.^[1]^ Cellular immunohistochemistry shows that HCOs stain positive for AFP, albumin, α-1 antitrypsin, and α-1 antichymotrypsin. To confirm HCO, it is necessary to differentiate it from other subtypes of AFP-producing tumors such as HCC, hepatoid yolk sac tumors (HYSTs), Sertoli-Leydig cell tumors, and dysgerminomas.^[3]^ Patients with HCC experiencing metastases to the ovary are rarely reported. Thus, it is necessary to exclude hepatic lesions clinically and radiologically and confirm bile component characteristics of HCC pathologically.

HYSTs are germ cell tumors that commonly develop in the young age group (average age of 22 years). It is difficult to differentiate HYST from HCO as the size of the tumor and serum level of AFP rapidly increases with tumor progression in both cases. Nevertheless, testing paraffin-1 negativity for germ cell component and immunohistochemical studies are useful for differentiating the two.^[3]^

The optimal approach for treatment of HCO remains unknown. In almost all cases, the patients received treatment similar to epithelial ovarian cancer, including maximal cytoreductive surgery, which would eliminate macroscopic disease such as hysterectomy, bilateral salpingo-oophorectomy, with or without lymph node dissection, and/or omentectomy. Most patients including our case (9 cases) have been treated with adjuvant chemotherapy regimens such as carboplatin and paclitaxel for ovarian cancer.^[3,7,8,12,14–17]^ Only three patients showed a good response without recurrence^[3,8,16]^ but the others recurred several months later, two of relapsed patients showed a short response but worsened during treatment.^[12,15]^

Three patients who underwent intraperitoneal chemotherapy with or without adjuvant chemotherapy^[13,14,19]^ did not show intraperitoneal recurrence. This suggests that intraperitoneal chemotherapy may be as effective as ovarian cancer with peritoneal dissemination.

Platinum and gemcitabine combination therapy, doxorubicin, and docetaxel were used as a secondary chemotherapy, but it did not show any good effect.

Recently, there have been attempts to treat HCO with sorafenib, a liver cancer targeted drug, but it was stopped after 2 months due to HCO progression.^[12]^

The most common metastatic sites of HCO are peritoneum, intraperitoneal lymph node intraabdominal visceras such as liver, spleen, and distant hematogenous metastasis is less common.^[19]^ Only one patient showed bone metastasis in the lumbar spine (L2) as in our case and was reported as alive after complete remission through radiation therapy without surgery.^[14]^ Of the two patients who received radiation therapy for bone metastasis, our case showed a decrease in AFP after radiation treatment, which could help predict the effectiveness of the treatment.

The reported data suggest that the prognosis of HCO is very poor, and 5 out of 9 patients died within 3 years.^[7,15,17,18]^ However, some reports showed that patients who have been diagnosed in stage 3 or 4 survived more than 2 years through surgery and chemotherapy.^[3,14,19]^ Pandey et al^[12]^ suggests that despite histopathological similarity between HCO and HCC, HCO should be treated similarly to epithelial ovarian cancer rather than HCC because HCO is biologically similar to ovarian cancer unlike HCC.

In this case, the diagnosis of HCO was confirmed using histopathologic and immunohistochemical staining. Abdominal ultrasound, abdominal CT, upper gastrointestinal endoscopy, colonoscopy, and finally, surgery were used to exclude other organ origins.

**Figure 4:**
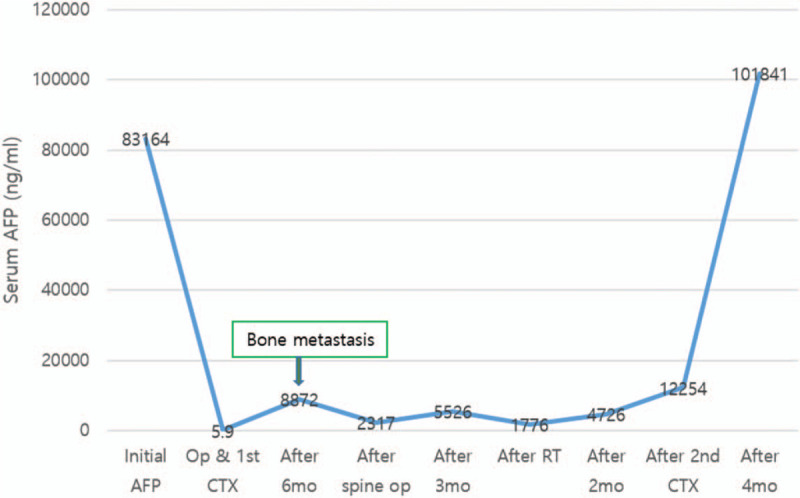
Levels of serum alpha-fetoprotein after treatment for HCO.

When compared to cases reported thus far, the patient was diagnosed with HCO at an early stage, FIGO stage IC and maintained a remission state for 6 months after the end of chemotherapy. However, the serum level of AFP increased, and bone metastasis was confirmed. Serum AFP level rapidly increased after multiple intraabdominal metastases. Thus, the serum level of AFP is useful as a prognostic marker for recurrence or metastasis evaluation (Fig. 4). Although the patient underwent radiation therapy and second-line chemotherapy, she showed a limited response and poor prognosis.

In conclusion, HCO is a malignant ovarian tumor with noted hepatoid features originating from the ovary. It has noted nonspecific symptoms and signs. Recommended treatments for HCO and ovarian cancer are similar and include debulking operation and chemotherapy. Despite rare incidence, HCO recurs commonly. In addition, it is often resistant to surgery and chemotherapy. HCO was evaluated in this study with limited data based on case report review. There is no established consensus on its histological or immunochemical staining patterns for diagnosis or treatment directions. Therefore, further case reviews and associated histological and molecular biologic studies are needed to establish optimal diagnosis and treatment for HCO.

## 4 Conclusion

HCO is a rare, malignant tumor of the ovary. HCO is generally associated with symptoms such as abdominal pain when it is over 10 cm in size, and it is most often diagnosed in the advanced stages. It is likely that serum AFP level may be useful as a prognostic marker for disease progression and recurrence. Treatment of ovarian cancer is limited and includes surgery and adjuvant platinum-based chemotherapy, and new therapeutic approaches such as target-based therapy and immunotherapy are needed.

## Acknowledgments

None of the authors had a conflict of interest with respect to these data reported. This paper was supported by funds from the Research Institute of Clinical Medicine of Jeonbuk National University-Biomedical Research Institute, Jeonbuk National University Hospital.

## Author contributions

**Conceptualization:** Won-Ku Choi, Chang-Yeol Yim, Na-Ri Lee.

**Data curation:** Won-Ku Choi.

**Formal analysis:** Won-Ku Choi, Chang-Yeol Yim.

**Investigation:** Dong-Hyu Cho.

**Resources:** Dong-Hyu Cho.

**Software:** Chang-Yeol Yim.

**Supervision:** Dong-Hyu Cho, Na-Ri Lee.

**Visualization:** Dong-Hyu Cho.

**Writing – original draft:** Won-Ku Choi.

**Writing – review & editing:** Na-Ri Lee.
